# Comparison of exhaled breath condensate pH using two commercially available devices in healthy controls, asthma and COPD patients

**DOI:** 10.1186/1465-9921-10-78

**Published:** 2009-08-24

**Authors:** Rembert Koczulla, Silvano Dragonieri, Robert Schot, Robert Bals, Stefanie A Gauw, Claus Vogelmeier, Klaus F Rabe, Peter J Sterk, Pieter S Hiemstra

**Affiliations:** 1 Department of Pulmonology, Leiden University Medical Center, the Netherlands; 2 Department of Pulmonology, Philipps University Marburg, Germany; 3 Department of Respiratory Medicine, Academic Medical Center, Amsterdam, the Netherlands

## Abstract

**Background:**

Analysis of exhaled breath condensate (EBC) is a non-invasive method for studying the acidity (pH) of airway secretions in patients with inflammatory lung diseases.

**Aim:**

To assess the reproducibility of EBC pH for two commercially available devices (portable RTube and non-portable ECoScreen) in healthy controls, patients with asthma or COPD, and subjects suffering from an acute cold with lower-airway symptoms. In addition, we assessed the repeatability in healthy controls.

**Methods:**

EBC was collected from 40 subjects (n = 10 in each of the above groups) using RTube and ECoScreen. EBC was collected from controls on two separate occasions within 5 days. pH in EBC was assessed after degasification with argon for 20 min.

**Results:**

In controls, pH-measurements in EBC collected by RTube or ECoScreen showed no significant difference between devices (p = 0.754) or between days (repeatability coefficient RTube: 0.47; ECoScreen: 0.42) of collection. A comparison between EBC pH collected by the two devices in asthma, COPD and cold patients also showed good reproducibility. No differences in pH values were observed between controls (mean pH 8.27; RTube) and patients with COPD (pH 7.97) or asthma (pH 8.20), but lower values were found using both devices in patients with a cold (pH 7.56; RTube, p < 0.01; ECoScreen, p < 0.05).

**Conclusion:**

We conclude that pH measurements in EBC collected by RTube and ECoScreen are repeatable and reproducible in healthy controls, and are reproducible and comparable in healthy controls, COPD and asthma patients, and subjects with a common cold.

## Background

The accessibility of the respiratory system compared with the internal organs provides a unique opportunity for non-invasive assessment of inflammation present in most respiratory diseases. Non-invasive techniques for analyzing inflammatory mediators present in lower airway secretions include the collection of induced sputum (IS) and exhaled breath condensate (EBC). EBC is a technique first described by Russian researchers in the early Eighties [1,2]. The use of EBC collection and analysis has several advantages: It is non-invasive, easy to use, allows repeated sampling, and is suitable for analysis of children and patients with severe disease on mechanical ventilation [3,4]. EBC can be collected by commercially available devices such as ECoScreen™ (Jaeger, Wuerzburg Germany), RTube™ (Charlottesville, Virginia, USA), as well as by self-made devices. Recently an ATS/ERS Task Force has published methodological recommendations regarding the use of EBC [5], but no recommendations regarding the device were presented, which probably reflects the lack of comparative studies.

Many studies have used assessment of EBC pH as a measure of airway acidity in association with airways inflammation [6,7]. Low EBC pH is found in patients with a variety of inflammatory lung disorders, including cystic fibrosis, COPD, asthma, as well as patients undergoing graft rejection following lung transplantation [6-9]. However, there is no consensus regarding collection and analysis of EBC for pH measurement, which is thought to be partly dependent on the collection device [10]. The aim of this study was to compare the results of two commercial devices for sampling EBC, ECoScreen and RTube. First, we assessed the between-day repeatability of this analysis for both devices in the healthy controls. Second, we analysed the agreement of ECoScreen and RTube by collecting EBC from each device once on the same day. Finally, we examined the between-group differences of EBC pH values obtained by these two devices in healthy controls (HC), asthmatics, COPD patients and patients with a cold.

## Methods

### Subjects

#### Healthy controls (HC)

Ten healthy volunteer controls (HC; non-smokers) between 23–54 years of age were included in the study. Asthma and COPD were ruled out by a negative history of respiratory symptoms.

#### COPD patients

Ten stable COPD patients recruited into the study met the following criteria: aged 52–67; ex- or current smoker with at least 10 pack-yrs and no history of asthma (Table 1). Furthermore, patients with COPD had irreversible airflow limitation, *i.e*. postbronchodilator forced expiratory volume in one second (FEV_1_) and FEV_1_/inspiratory vital capacity <90% confidence interval of the predicted value, FEV_1 _1.3 L and >20% of the predicted value [11], as well as one or more of the following symptoms: chronic cough, chronic sputum production, or dyspnoea on exertion. Postbronchodilator lung function was measured in COPD patients for disease staging according to GOLD criteria [12], and all patient met GOLD stages 1 and 2 (Table 1). Patients had not used a course of steroids during the 3 months prior to randomization, and had not received maintenance treatment with inhaled or oral steroids during the previous 6 months.

**Table 1:** Clinical characteristics of the study population

	Healthy control	Asthma	COPD	Cold
No. of patients	10	10	10	10
Age (y)	34 ± 11.2	25.1 ± 5.9	60.8 ± 5.4	29.6 ± 6.4
Sex (male/female)	5/5	1/9	8/2	7/3
Smoker/non smoker	0/10	0/10	7/3*	1/9
FEV_1 _pre broncho. (% pred.)	ND	99.9 ± 7.7	70 ± 14.8	ND
FEV_1 _post broncho. (% pred.)	ND	111.9 ± 9.2	8.5 ± 13.3	ND
FVC (% pred.)	ND	109 ± 8.1	68 ± 11.6	ND

* = ex smoker, ND = Not Determined; Values are expressed as means ± SDs

%pred. =% predicted, pre broncho = pre bronchodilator; post broncho = post bronchodilator

#### Asthma patients

Ten non-smoking male and female patients with mild intermittent asthma according to the Gina workshop report [13], aged 21–43, were included in this study. They had episodic chest symptoms and showed a baseline forced expiratory volume in 1 second (FEV_1_) ≥ 70% of predicted, a provocative concentration of methacholine chloride causing a 20% fall in FEV_1 _(PC_20_) < 8 mg/ml, and positive skin prick tests (SPT). The patients were clinically stable, only used *β*_2_-agonists on demand, and had no history of recent respiratory tract infection within 4 weeks from the start of the study. Corticosteroid therapy was not allowed within 8 weeks prior to screening, nor during the study.

#### Patients with a cold

Ten otherwise healthy subjects with early onset of a common cold with clinical symptoms like cough, nasal discharge, sneezing, stuffy nose, malaises, chills or fever, according to the definition of Lemanske et al. [14] were scheduled for a visit less than 12 hours after onset of the symptoms. All participants met the following criteria: aged 24–47; one patient smoked, but had less than 10 pack years (py); none of the patients had a history of asthma (Table 1).

The Medical Ethics Committee of the Leiden University Medical Center granted approval for the study. All subjects gave written informed consent prior to the study.

### Design

The healthy controls (HC) visited the laboratory on two days. On each day EBC was collected once by ECoScreen and once by RTube in random order and with a 10 min interval. The second visit was scheduled within 5 days after the first in order to assess repeatability.

The patients with COPD, asthma or a cold had a single study visit, at which EBC was also obtained twice, once with each device (RTube vs. ECoScreen). Randomization for the device was performed as described above.

### Measurements

Prior to use, all parts of the collection devices that came into contact with the EBC were rinsed with double deionized water to remove possible contaminants, and were air dried before use. In pilot experiments, we observed that drinking coffee or smoking shortly (within one hour) before EBC collection affected pH levels (data not shown). Therefore, subjects were asked to refrain from eating, drinking coffee and smoking for at least 2 hours before EBC collection. The RTube sleeve was cooled to -20°C for at least 1 h before use. The ECoScreen was cooled to -10°C as prescribed by the manufacturer.

EBC was collected during 10 min tidal breathing with the subjects wearing a noseclip. After collection, 200 μl μl of the EBC sample was immediately transferred to polypropylene tubes, degassed with argon gas (purity > 99%) at a flow rate of 350 ml/min (= 6 ml/s) bubbling through the EBC sample. pH analysis was performed with a thin and sensitive glass electrode and pH meter (Beckman, USA). In order to assess the effects of degasification on fresh and frozen samples, pH measurements after various periods of degasification with argon gas on fresh EBC samples were compared to those in EBC samples that were stored at -20°C for 7 days. To this end, EBC was collected from 5 additional healthy controls and immediately aliquoted into samples of 200 μl after collection. All subsequent analysis were performed on fresh samples after at least 20 min of degasification.

### Statistical analysis

Analysis of repeatability and reproducibility was done according to Bland Altman[15,16]] The reproducibility of the pH values by ECoScreen and RTube was assessed from the duplicate measurements. Similarly, the between-day repeatability for both systems was analysed from the two readings of RTube and ECoScreen in the HC group. To study the reproducibility of EBC pH for ECoScreen and RTube, we calculated the mean of the duplicate measurements by each method on each subject and used these pairs of means to compare the two methods. In order to determine the limits of agreement, we first calculated the standard deviation of the differences between the averaged measurements for the two devices according to Bland-Altman. The respective variance is given as mean of the 2 within-subject variances, s_w1_^2 ^and s_w2_^2^, added to the variance of the differences between the within-subject means s_d_^2^[15,16]. Differences between pH values in subjects from different groups were first explored using ANOVA tests; subsequently differences between subject groups were analyzed using a post-hoc Bonferroni multiple comparisons test. Differences at p-values < 0.05 were considered significant.

## Results

During the whole sampling period, no adverse effects were noted in any of the study groups, except for one mild form of hyperventilation on the ECoScreen in the HC group. After collection, 200 μl of each sample was immediately removed for pH analysis.

### Effect of duration of degasification on fresh and frozen EBC samples

**Figure 1:**
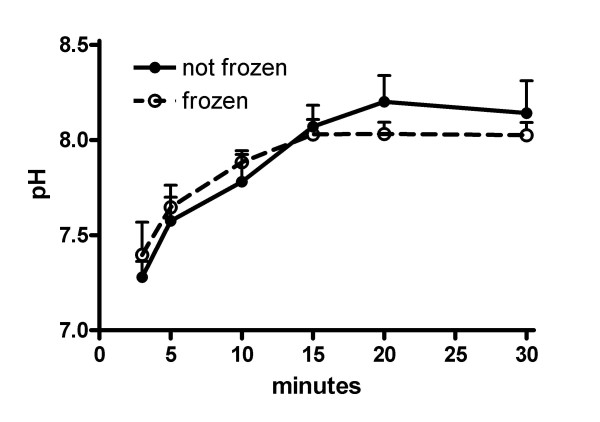
**Effect of duration of degasification on fresh and frozen EBC samples**. EBC samples collected by RTube were obtained from 5 healthy controls, and either used fresh or after storage at -20°C (frozen). Prior to pH analysis, EBC samples were de-aerated using argon gas for various periods of time. Results show mean ± SD.

Degasification of EBC samples using argon gas removes CO_2 _from the sample, thus allowing standardization of measurements between EBC samples that may have different CO_2 _baseline levels. We therefore first determined the optimal time of gas standardization with argon gas, using EBC samples collected by RTube from 5 healthy controls that were immediately aliquoted after collection. In these experiments we also compared freshly collected EBC samples to those that were stored immediately after collection for 7 days at -20°C and thawed shortly before use. The results show that after 20 minutes the pH values stabilize, and demonstrate no essential differences between fresh and frozen samples (Figure 1).

### Between-day repeatability of EBC pH in healthy controls for the two devices

Between-day repeatability of the two methods was assessed in order to compare the performance of both devices.

#### EBC pH for ECoScreen (Figure 2a)

The mean difference between the measurements on both study days was 0.016 (SD = 0.18), and not significant (p = 0.785). The within-subject standard deviation, s_w_, for EBC pH assessed on samples collected by ECoScreen was 0.15. The repeatability coefficient was 0.416, indicating that two readings by the same method will be between – 0.42 and 0.42 for 95% of the subjects.

**Figure 2:**
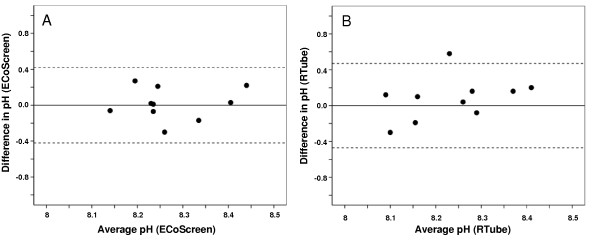
**Bland-Altman plot showing within-subject repeatability of pH in EBC collected by ECoScreen (Figure 2A) and RTube (Figure 2B) from 10 healthy subjects on two different days**. Horizontal lines show the mean and 95% confidence interval.

#### EBC pH for Rtube (Figure 2b)

The mean differences between the measurements on both study days by RTube was -0.08 (SD = 0.24), and not significant (p = 0.33). The within-subject standard deviation, s_w_, for EBC pH assessed on samples collected by RTube was 0.17. The repeatability coefficient was 0.47, indicating that two readings by the same method will be between – 0.47 and 0.47 for 95% of the subjects.

Comparing the two collection devices regarding the between-day repeatability in assessing EBC pH on two different days showed that ECoScreen and RTube show no systematic difference and have the same variability.

### Agreement between ECoScreen and RTube

**Figure 3:**
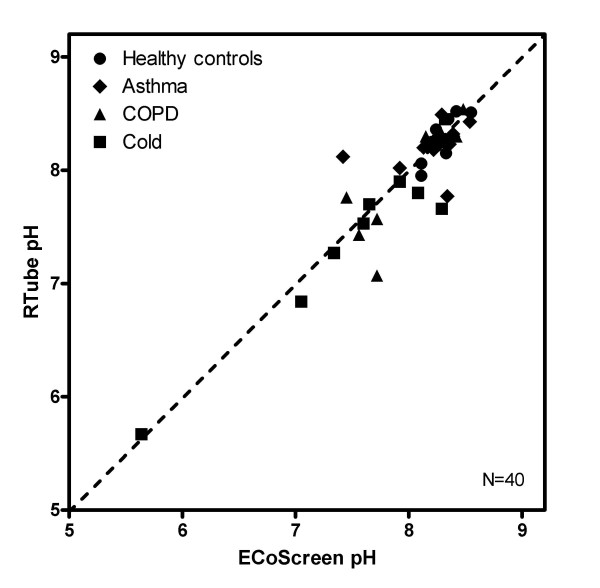
**Comparison of pH in EBC obtained by either RTube or ECoScreen in healthy controls (HC), asthma, COPD and cold patients (n = 40)**. The dashed line is the line of identity.

The values of consecutive samples from both collection devices in healthy controls (n = 40) were compared regarding the pH assessment. The mean pH of all samples collected by RTube for all groups was 7.99 (SD 0.56), and for samples collected by ECoScreen it was 8.03 (SD 0.53). There was no significant difference between mean RTube pH and mean ECoScreen pH (p = 0.754) (Figure 3).

**Figure 4:**
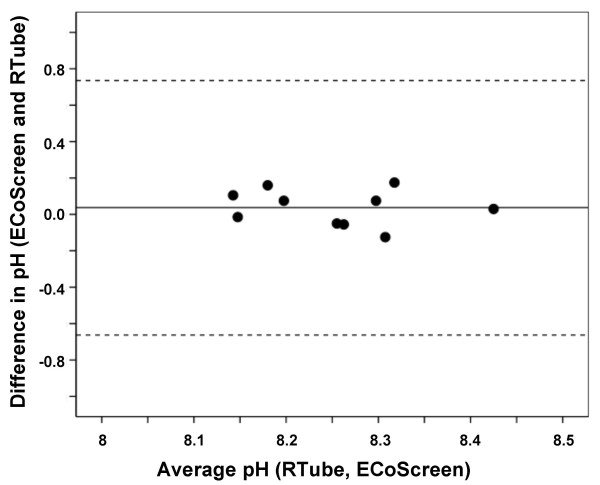
**Bland-Altman plot showing good agreement between pH values in EBC collected from 10 healthy subjects using ECoScreen and RTube on two different days**. Horizontal lines show the mean and 95% confidence interval.

We next performed a Bland-Altman analysis on the data from the healthy controls (n = 10) to assess the reproducibility of the measurement using both devices in these subjects (Figure 4). The mean difference between the within-subject means of ECoScreen and RTube EBC pH was only 0.0375 (SD = 0.1) and non-significant (p = 0.258; paired t-test). The 95% limits of agreement was 0.0375 ± 0.70 (mean ± 2SD). These data show an excellent reproducibility between pH values in EBC samples collected by both devices based on consecutive measurements in healthy controls.

### EBC pH in patients with asthma, COPD and common colds

#### Reproducibility

**Figure 5:**
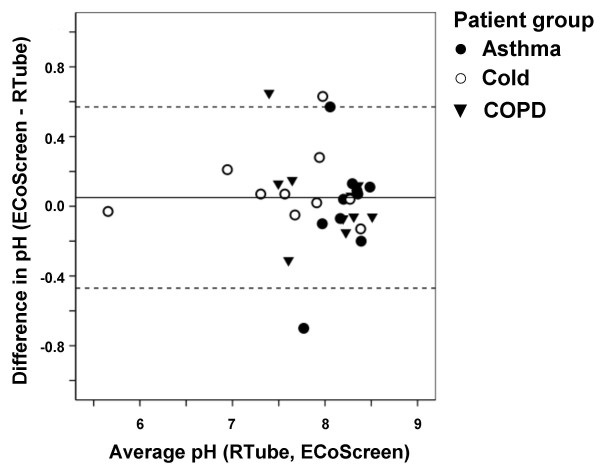
**Bland-Altman plot showing good agreement between pH values in EBC collected by ECoScreen and RTube from patients with asthma, COPD or a cold (n = 10 for each group)**. Horizontal lines show the mean and 95% confidence interval.

After showing repeatability and reproducibility of EBC pH for ECoScreen and RTube, we next analyzed the reproducibility of EBC pH values obtained with the two collection devices in different patient groups (Figure 5). The mean of the differences ECoScreen-RTube in the values from all three patient groups was 0.05 (SD = 0.26) and non-significant (p = 0.302; paired t-test), resulting in a 95% limit of agreement of 0.05 ± 0.52. These results show that both devices show good reproducibility, not only in healthy controls, but also in patients.

#### Comparison of pH values

**Figure 6:**
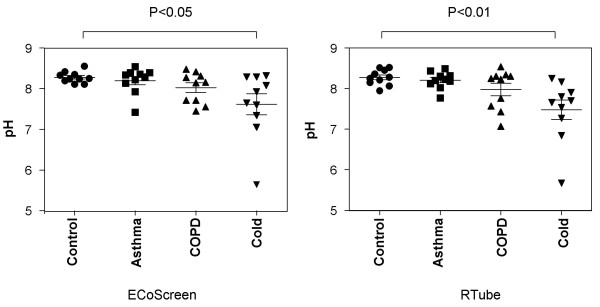
**Comparison of EBC pH values obtained by ECoScreen (left panel) or RTube (right panel) in healthy controls, and patients with asthma, COPD or a cold (n = 10 per group)**. Using both devices, pH values in patients with a cold were significantly lower than in healthy controls, whereas the other patient groups did not show a difference.

The mean pH in EBC collected by RTube was 8.27 (SD 0.19) in HC, 8.20 (SD 0.2) in asthma patients and 7.97 (SD 0.48) in COPD patients. The mean pH in the cold group (pH 7.56, SD 0.77) was significantly lower compared to the HC group (p < 0.01) (Figure 6). Data from the ECoScreen (p < 0.05) showed comparable results.

## Discussion

The results from the present study show that EBC pH values can be well assessed both using ECoScreen and RTube, with good repeatability and reproducibility. First, the results show excellent repeatability in healthy controls for both devices when studied within a period of 5 days. The order in which the two devices were used did not make any difference (data not shown). Second, comparison of pH values obtained by EBC analysis following collection by ECoScreen and RTube, shows good reproducibility not only in healthy controls, but also in patients with COPD, asthma or a cold. Third, our study shows comparable results using freshly collected samples and samples that were divided into aliquots and stored frozen prior to analysis. Comparison of pH values between the different groups showed that values in patients with a cold were significantly lower than in healthy controls, but no other differences were noted between patient groups. These data show that pH measurements in EBC collected by RTube and ECoScreen are repeatable and reproducible in healthy controls, and reproducible in COPD and asthma patients, and subjects with a common cold.

Several studies have demonstrated the simplicity of obtaining EBC in healthy subjects and various patient groups [10,17,18]. The study design and the processing of the EBC probes in those studies differed in various points compared to ours. In the present study healthy controls, as well as patients with asthma, COPD or a cold were studied to obtain a wide range of pH values and to address methodological questions in relevant patient groups. Our results on the performance of the RTube and ECoScreen for pH analysis of EBC in healthy controls extend those of Soyer *et al *[10], who showed that RTube and ECoScreen provide nearly identical pH results in 30 healthy controls. The values reported by Soyer *et al *in healthy controls [ECoScreen 7.55 (6.88–7.90) *vs *RTube 7.54 (7.09–7.93), *p *= 0.419] were about 0.51 lower than the values we observed in the present study [10]. This may be explained by the difference in the duration of degasification, which was 10 minutes in the study by Soyer, and 20 minutes in the present study. The longer degasification period in our study was based on results from experiments showing that stabilization of the pH measurement required 20 minutes degasification with argon.

Regarding the comparability of the two devices in asthmatics, a previous study showed that pH values in EBC collected by RTube and ECoScreen are comparable (mean difference 0.28) in a small group of asthmatic children aged 14–22 (mean age 14 years). The median pH for the RTube was 8.07 ± 1.23, which is fairly close to the values we found for stable asthmatics [19]. In contrast, Prieto and co-workers reported significantly higher pH values in EBC obtained by ECoScreen compared to the RTube in healthy controls, asthmatic and allergic subjects before and after deaeration [17]. This different result may be explained by the fact that this study differed from our study by *i*. the small sample size (asthmatics: n = 10, allergic rhinitis: n = 7, HC: n = 6); *ii*. the pH-meter and calibration procedure used, as well as other local conditions such as temperature; and *iii*. the fact that no nose clips were used during collection, allowing air to pass the upper airways by inspiration, thus possibly influencing the exhaled breath values in patients who breath additionally through the nose. Indeed, in another study comparing collection with and without nose clips in COPD subjects, higher pH values were observed in those wearing nose clips [8]. Finally, in Prieto's study the degasification time of the sample was only 8 min. Our study therefore confirms the reproducible pH values in EBC from healthy controls collected using both devices, and adds to these the repeatability of the results in both devices in healthy controls, and the reproducibility of results obtained with both devices in patients with inflammatory lung diseases or an acute cold. Whereas degasification is known to cause a gradual increase in pH until a stable pH is reached, there is no consensus on the duration of this degasification [5]. We speculate that the prolonged and optimized degasification time used in the present study has contributed to the marked repeatability and reproducibility of both devices in the present study.

The second observation of our study was the high between-day repeatability of both devices. This was also found very recently for healthy controls and mild to severe asthmatics. In contrast to our work, in this study by Accordino *et al*. EBC was collected with a home made apparatus and the sample degasification time with argon was three minutes which is much shorter compared to the optimized time period used in our study [20]. Our observation on high between-day repeatability is also confirmed by results in a study by Vaughan, Hunt and co-workers who reported a small mean coefficient of variation, based on consecutive measurements using the RTube for obtaining EBC [4]. One important explanation for the good repeatability appears to be the prolonged degasification of the sample [4], which appears to decrease variability. Hunt and co-workers adopted the procedure of removing CO_2 _by flushing the samples with argon, an inert gas, thereby removing CO_2 _from the EBC solution. The initial pH values in the non-de-aerated EBC from healthy controls were indeed lower than when de-aerated, suggesting that the end-tidal CO_2 _of about 40 mmHg in the exhaled air decreases pH values. We observed the most stable pH values after at least 20 min degasification. Complete removal of CO_2 _after this degasification period was confirmed by CO_2 _analysis using a blood gas device (Radiometer Copenhagen; data not shown). In contrast, recently published work from the Horvath group showed that the use of a constant CO_2 _concentration of 5.33 kPa (40 mmHg), which is the physiological alveolar CO_2 _pressure, to treat EBC samples resulted in the most reproducible pH condensate values [21]. However, they observed no pH correlation in EBC between RTube and ECoScreen using this procedure [22]. Based on the ATS/ERS statements for EBC, so far no clear recommendation exists regarding the need for degasification of EBC prior to analysis, or the use of a special gas [5].

We included stable asthmatics and COPD GOLD stage 1 and 2. Comparing the asthmatic and COPD patients with healthy controls, we did not find significantly different pH values in these patient groups which is in line with another report [20]. In contrast, lower values for EBC pH values were observed in other studies for asthma and COPD patients [7,8,20]. One possible explanation for this difference is that we measured symptom-free stable asthmatics, whereas Hunt *et al *studied unstable asthma subjects who were admitted to the hospital with dyspnoea [7]. Obviously, the lower pH in the EBC of these patients may be explained by increased pulmonary inflammation in unstable asthmatics. COPD subjects with GOLD grade 2 were investigated in the repeatability study from Borrill and colleagues [8]. They found the pH values in COPD subjects to be 0.6 log lower compared to the healthy controls which was statistically significant. In our COPD group, we included 5 patients with GOLD stage 1, and 5 with GOLD stage 2, indicating that Borrill studied more severe COPD patients which is likely reflected by the lower pH [8]. In line with this, we found slightly lower pH levels for GOLD 2 compared with GOLD 1 (data not shown). It is unlikely that the higher age of the COPD patients compared to the other study groups in our study explains the fact that we did not find a lower pH in these patients, since recently it was found that healthy subjects aged in the 60–80 age range have a slightly lower EBC pH [23]. Whereas we did not find differences between asthma or COPD patients, the patients with a cold in the present study had a lower pH compared to healthy controls. These patients were diagnosed based on the definition by Lemanske [14], and studied these patients within the first 12 hrs after onset of symptoms.

Our results show that EBC pH measurements have very limited potential in discriminating between healthy controls, and patients with mild asthma or COPD that are clinically stable. However, it may have potential in conjunction with other disease markers to discriminate patients. Furthermore, our observation in patients with a cold suggests that it may also be useful in monitoring asthma and COPD patients during infectious episodes, but this clearly requires further investigation. Decreased pH values in EBC in patients with inflammatory diseases like asthma and COPD have been shown by several study groups. However, this is the first comparison of two commercial devices in healthy controls and patients with asthma, COPD and colds regarding the measurement of pH, including degasification with argon, and showing a small interday and interdevice variability. Further experiments are necessary to obtain information about the repeatability and reproducibility of EBC for other volatile and non-volatile compounds using RTube or ECoScreen.

## Conclusion

EBC collection and pH analysis is extremely simple to perform, non-invasive, inexpensive and reproducible. Therefore, it is well-suited for non-invasive analysis in longitudinal follow-ups of individual patients, and patients can be provided with a portable device for collection of EBC at home. Based on our observations and strict procedures, both commercial devices provide equal results with respect to assessment of pH in EBC. This strong reproducibility enables the interchangeable use of these devices, if this would be required based on e.g. logistic considerations. We suggest a longer degasification time of at least 20 min to obtain stable pH values, since this seems to decrease variability of the measurement.

## Competing interests

The authors declare that they have no competing interests.

## Funding

This study was supported in part by a grant from Glaxo Smith Kline (GSK) and the German Respiratory Association (Deutsche Atemwegsliga) prize.

## List of abbreviations

COPD: chronic obstructive lung disease; EBC: exhaled breath condensate; HC: healthy controls; ND: Not Determined; post broncho: post bronchodilator; pre broncho: pre bronchodilator; Py: (cigarette) pack years; SPT: skin prick test; %pred.: % predicted.

## Authors' contributions

RK, PH and PS contributed to the design, conception, analysis and interpretation of the study. RK performed the experiments and drafted the manuscript. SD helped in acquiring patient data. PH, PS, CV, RB, RS and KR were involved in drafting and revising the manuscript. SG carried out the logistics and appointed all included study patients. RS contributed to setting up the EBC system. All authors read and approve the final manuscript.
